# Energetic dysfunction in sepsis: a narrative review

**DOI:** 10.1186/s13613-021-00893-7

**Published:** 2021-07-03

**Authors:** Sebastien Preau, Dominique Vodovar, Boris Jung, Steve Lancel, Lara Zafrani, Aurelien Flatres, Mehdi Oualha, Guillaume Voiriot, Youenn Jouan, Jeremie Joffre, Fabrice Uhel, Nicolas De Prost, Stein Silva, Eric Azabou, Peter Radermacher

**Affiliations:** 1 U1167 - RID-AGE - Facteurs de Risque et Déterminants Moléculaires des Maladies Liées au Vieillissement, University Lille, Inserm, CHU Lille, Institut Pasteur de Lille, F-59000 Lille, France; 2 Centre AntiPoison de Paris, Hôpital Fernand Widal, APHP, 75010 Paris, France; 3 Faculté de pharmacie, UMRS 1144, 75006 Paris, France; 4 Université de Paris, UFR de Médecine, 75010 Paris, France; 5 Medical Intensive Care Unit, Lapeyronie Teaching Hospital, Montpellier University Hospital and PhyMedExp, University of Montpellier, Montpellier, France; 6 Médecine Intensive Réanimation, Hôpital Saint-Louis, AP-HP, Université de Paris, Paris, France; 7 INSERM UMR 976, Hôpital Saint Louis, Université de Paris, Paris, France; 8 PhyMedExp, University of Montpellier, Montpellier, France; 9 Pediatric Intensive Care Unit, Necker Hospital, APHP, Centre - Paris University, Paris, France; 10 Service de Médecine Intensive Réanimation, Sorbonne Université, Assistance Publique - Hôpitaux de Paris, Hôpital Tenon, Paris, France; 11 Service de Médecine Intensive Réanimation, CHRU Tours, Tours, France; 12 Faculté de Médecine de Tours, INSERM U1100 Centre d’Etudes des Pathologies Respiratoires, Tours, France; 13 Department of Anesthesia and Perioperative Care, University of California, San Francisco, CA 94143 USA; 14 Réanimation médico-chirurgicale, Université de Paris, Assistance Publique - Hôpitaux de Paris, Hôpital Louis Mourier, Paris, France; 15 Service de Réanimation Médicale, Hôpital Henri Mondor, Assistance Publique-Hôpitaux de Paris, Cedex 94010 Créteil, France; 16 Réanimation URM CHU Purpan, Cedex 31300 Toulouse, France; 17 Toulouse NeuroImaging Center INSERM1214, Cedex 31300 Toulouse, France; 18 Clinical Neurophysiology and Neuromodulation Unit, Departments of Physiology and Critical Care Medicine, Raymond Poincaré Hospital, AP-HP, Inserm UMR 1173, Infection and Inflammation (2I), University of Versailles (UVSQ), Paris-Saclay University, Paris, France; 19 Institut für Anästhesiologische Pathophysiologie und Verfahrensentwicklung, Universitätsklinikum, Ulm, Germany

**Keywords:** Energetic dysfunction, Metabolism, Mitochondria, Mitochondrial dysfunction, Organ dysfunction, Sepsis, Septic shock, Infection

## Abstract

**Background:**

Growing evidence associates organ dysfunction(s) with impaired metabolism in sepsis. Recent research has increased our understanding of the role of substrate utilization and mitochondrial dysfunction in the pathophysiology of sepsis-related organ dysfunction. The purpose of this review is to present this evidence as a coherent whole and to highlight future research directions.

**Main text:**

Sepsis is characterized by systemic and organ-specific changes in metabolism. Alterations of oxygen consumption, increased levels of circulating substrates, impaired glucose and lipid oxidation, and mitochondrial dysfunction are all associated with organ dysfunction and poor outcomes in both animal models and patients. The pathophysiological relevance of bioenergetics and metabolism in the specific examples of sepsis-related immunodeficiency, cerebral dysfunction, cardiomyopathy, acute kidney injury and diaphragmatic failure is also described.

**Conclusions:**

Recent understandings in substrate utilization and mitochondrial dysfunction may pave the way for new diagnostic and therapeutic approaches. These findings could help physicians to identify distinct subgroups of sepsis and to develop personalized treatment strategies. Implications for their use as bioenergetic targets to identify metabolism- and mitochondria-targeted treatments need to be evaluated in future studies.

**Supplementary Information:**

The online version contains supplementary material available at 10.1186/s13613-021-00893-7.

## Introduction

Sepsis is a well-recognized, worldwide healthcare issue defined as life-threatening organ dysfunction resulting from a dysregulated host response to an infection [1–5]. This response is generally characterized by an acute and massive release of stress hormones, leading to an overwhelming production of energy substrates in the form of glucose, fatty acids (FA), amino acids and lactate. The most severe cases tend to exhibit elevated levels of plasmatic glucose [6], triglycerides [7], and lactate [8–13], while hypoglycaemia in combination with elevated lactataemia has been associated with poor outcomes [6].

**Fig. 1:**
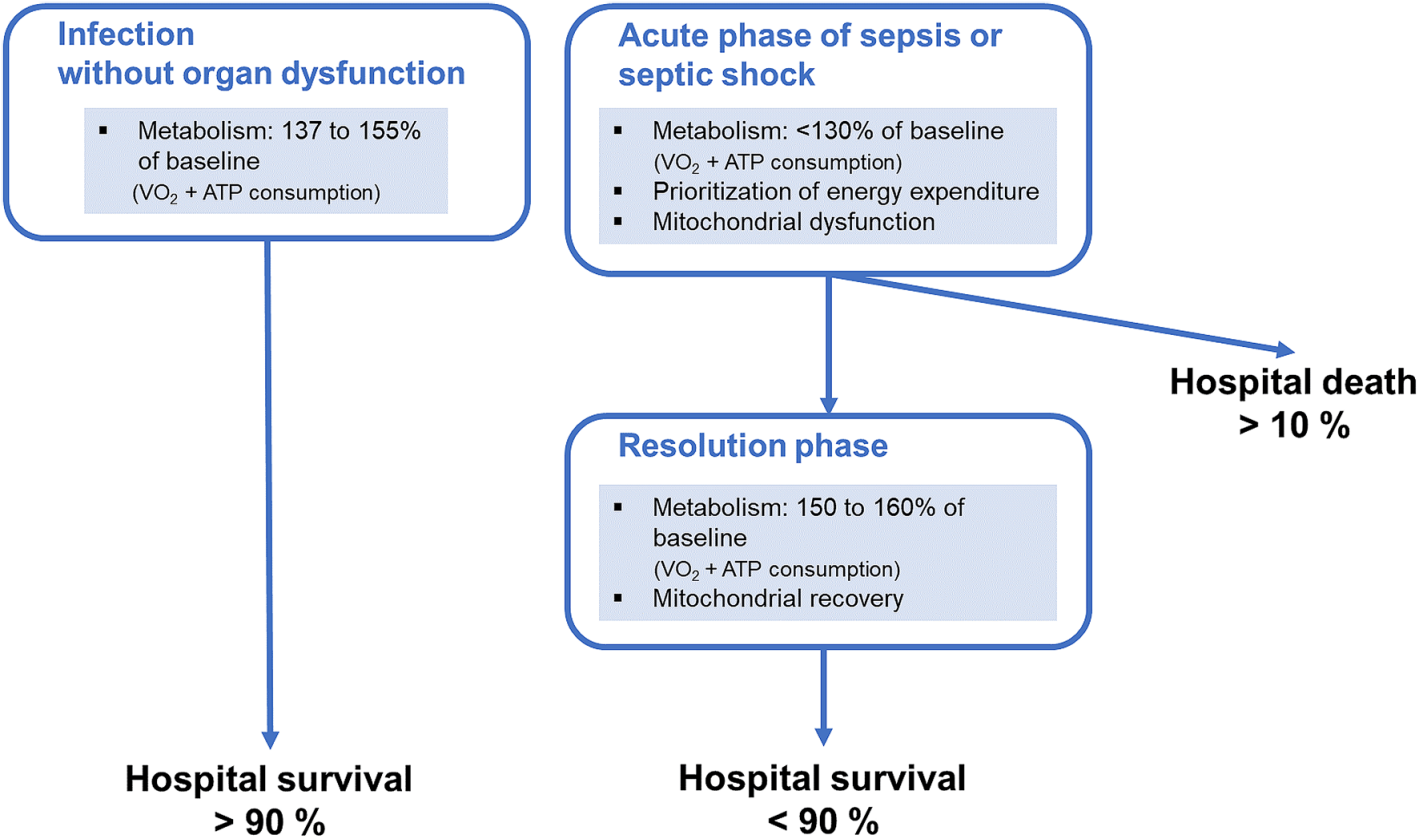
Different metabolic features of patients hospitalized for infection. *ATP* adenosine triphosphate, *VO*_*2*_ oxygen consumption

Moreover, different features of oxygen (O_2_) consumption (VO_2_) characterize patients with infections according to their clinical severity (Fig. 1). In infected patients without organ dysfunction(s) and healthy humans treated with a non-lethal dose of endotoxin, systemic VO_2_ and resting metabolic rate are enhanced by 37–55% compared with their basal metabolism [14–16]. Conversely, patients with sepsis or septic shock seem to display a less-pronounced increase in the metabolic rate from baseline (< 30%) [14, 17, 18]. This metabolic attenuation has been described in patients admitted to intensive care units (ICU) for sepsis who displayed no, or only small, increases in VO_2_ and energy expenditure [14] in the absence of (tissue) hypox(aem)ia [19]. This diminished increase in these two parameters is related to the severity of sepsis [14, 17, 20], while a lack of responsiveness to the increase of systemic O_2_ delivery is also associated with poorer outcome [21, 22]. The precise mechanism responsible for this attenuation of metabolic function is still a matter of debate and may be attributable to either a lack of adequate energy supply, to oxidative and nitrosative stress-related damage, or to a reprioritization of adenosine triphosphate (ATP) consumption.

Mitochondrial dysfunction participates in the pathophysiology of sepsis and is associated with patients’ outcomes [23]. The key role of mitochondria in cellular homeostasis hints at a mechanistic explanation for the link between mitochondrial dysfunction and sepsis-related organ dysfunction. While the mitochondrial capacity for generating cellular ATP is decreased in sepsis, mitochondrial dysfunction is not associated with significant organ necrosis in human septic shock [24–26]. The shift in energy expenditure during sepsis, from anabolic functions like protein and nucleic acid synthesis to those essential for short-term survival such as the maintenance of ionic homeostasis [27] (i.e. Na + /K +- and Ca2 + -ATPase pumps), may preserve cells’ integrity despite their dysfunction, and retain the potential for rapid recovery from organ failure among survivors [24, 26]. During the recovery phase of hospital survivors, systemic VO_2_ and resting metabolic rate are enhanced by 50–60% compared with normal basal metabolism [14, 28, 29]. A patient‘s ability to adequately evolve from mitochondrial dysfunction and metabolic attenuation to mitochondrial recovery and hypermetabolism is a potentially important mechanism in determining recovery during their hospital stay [2, 30].

The objectives of this review are (1) to present an integrated view of the main changes in substrate utilization by cells during sepsis; (2) to discuss the significance of mitochondrial metabolism in the course of sepsis-induced organ dysfunction; and (3) to highlight the pathophysiological relevance of energy metabolism in specific examples of sepsis-related immunodeficiency, cerebral dysfunction, cardiomyopathy, acute kidney injury (AKI), and diaphragmatic failure.

## Substrate utilization for cellular energy during sepsis

Mizock has highlighted the “metabolic criteria” of stress; i.e. hypermetabolism associated with increased VO_2_, protein catabolism leading to increased urinary nitrogen loss, and insulin resistance [31]. Altered glucose metabolism comprises the key manifestations of this metabolic response, namely increased endogenous glucose production [32, 33] (unless glucose and/or insulin are exogenously supplied [33, 34]), in particular in patients with septic shock requiring catecholamine support [35–38] and impaired glucose oxidation [33, 34]. Consequently, the predominant bedside laboratory findings are hyperglycaemia and hyperlactataemia. Although considered as a physiological stress response [31], this hyperglycaemia has marked deleterious side effects in and of itself—e.g. oxidative stress [39]—and may aggravate organ injury [40, 41]. While hyperlactataemia originates mainly from peripherally released lactate, hyperglycaemia arises from the hepatic [16] (and, during adrenergic stimulation, renal) [38, 42, 43] uptake and conversion of both lactate (i.e. the Cori cycle) and glucogenic amino acids (in particular alanine) to glucose [44, 45]; i.e. glucose formation from gluconeogenesis rather than glycogenolysis [32]. Alanine, can also directly enter the Krebs cycle with α-ketoglutarate via alanine aminotransaminase to form glutamate and pyruvate. Nevertheless, in addition to the impact of insulin resistance, hyperglycaemia can be regarded as a mirror of the metabolic capacity of the gluconeogenic tissues, i.e. the periportal hepatocytes, where the highly O_2_-dependent metabolic pathways such as gluconeogenesis and ureagenesis are located [46], and the kidney [42, 43]. Early studies described impaired hepatic glucose release, despite increased organ blood flow and O_2_ uptake, in “bacteraemic burn patients with complications” [47]: it is a common clinical observation that the absence of hyperglycaemia upon catecholamine infusion and/or a sudden reduction of the amount of insulin required to achieve normoglycaemia often indicates impending liver failure. In cases of hepatic failure, the liver can no longer completely take up the circulating glucogenic amino acids. Since ureagenesis (again located in the periportal hepatocytes) is also impaired under these conditions, hepatic “detoxification” is compromised and ammonia blood levels will rise in consequence [48].

The hyperlactataemia mentioned above must not be confused [49, 50] with ischaemia-induced hyperlactataemia, which is associated with metabolic acidosis resulting from anaerobic glycolysis due to the imbalance between O_2_ supply and demand in tissues [51]. In contrast, sepsis-associated hyperlactataemia has a component that is not due to “hypoxia” (i.e. impaired tissue O_2_ supply [51]) or “dysoxia” (i.e. disturbed cellular O_2_ utilization [52]), but instead originates from adrenergic stimulation and Na^+^/K^+^-ATPase activation [53]. Perturbed O_2_ utilization is at least partly due to increased superoxide anion (O^2−^) generation within mitochondria, and the subsequent impairment of electron transfer within the mitochondrial chain [54]. The O^2−^ can react with nitric oxide (NO) to form the even more toxic peroxynitrite (ONOO^−^), which causes protein nitration detectable by increased nitrotyrosine concentrations and/or desoxyribonucleic acid (DNA) damage [55–57]. The latter will activate poly-ADP-ribose polymerase (PARP) to provide DNA repair, but this is a high-energy process and potentially contributes to metabolic breakdown and consequent organ failure [55]. In the context of enhanced O^2−^ production, coenzyme Q10 (CoQ10—a component of the electron transport chain with antioxidant properties) plays a crucial role. Indeed, blood levels of CoQ10 are reduced in patients with sepsis or septic shock and can be restored by exogenous CoQ10 supplementation [58, 59]. Lactate released from the skeletal muscles can assume major importance in sepsis-induced, “non-dysoxic” hyperlactataemia, even in the presence of small differences in arterio-venous lactate content [50], because the contribution of muscle to total body mass is large and the vast majority of muscle cell adrenoceptors are β_2_-receptors [49, 50]. In addition, the lung can become a “lactate producer” [60, 61], whereas the splanchnic region remains an overall “lactate consumer” [35–38, 62] due to the hepatic lactate uptake and conversion to glucose [63]. Immune cell metabolic switching from oxidative phosphorylation (OXPHOS) during the quiescent state to aerobic glycolysis upon activation (see below and the glossary in Additional file 1) [64–66] also contributes to lactate release to the blood [67]; this effect is, however, quantitatively less important due to the limited total white blood cell mass. Finally, this sepsis-associated hyperlactataemia has been referred to as an adaptive phenomenon [68], which may even facilitate lactate oxidation and bioenergetic efficiency in the brain and heart [69]. The release of counter-regulatory hormones and the formation of pro-inflammatory cytokines are the main reasons for sepsis-associated metabolic disturbances [70]. Clearly, the available human data originate from studies investigating human endotoxaemia [16], but the hemodynamic, cytokine and metabolic response to endotoxin infusion in volunteers closely resembled that in patients with sepsis or septic shock [71, 72]. In addition, the so-called “triple-hormone infusion” [73, 74] allows for modelling of the metabolic response pattern, thus confirming the crucial role of the counter-regulatory hormones. This metabolic condition resembles starvation and is characterized by the breakdown of protein, carbohydrate and fat reserves [75]. Sepsis-related alterations of fat metabolism—resulting from the hormonal conditions mentioned above—are characterized by upregulation of lipolysis in white adipose tissue [16], while free FA β-oxidation may nevertheless be decreased [75]: the lipolysis-related rise in free FA blood concentrations triggers the expression of the peroxisome proliferator-activated receptor (PPAR)-α, which in turn activates ketogenesis and β-oxidation. However, sepsis may be associated with PPAR-α downregulation, so that the available free FA may not be adequately metabolized via β-oxidation, resulting in free FA accumulation [75]. Metabolomic analysis has demonstrated that this metabolic deficit seems to be due to impaired acylcarnitine-dependent FA transport from the cytosol into the mitochondrion [76]. Sepsis-related cardiomyopathy [77], but also hospital mortality has, in part, been associated with this impaired FA oxidation [76]. Free FA accumulation may cause “lipotoxicity”, characterized by organ liver, kidney, and heart “steatosis” and, ultimately, mitochondrial injury [75]. Catecholamines assume particular importance for metabolic disturbances: all catecholamines with β-agonist properties induce “metabolic stress” [78], the extent of which relates to the substance-specific β-adrenergic potency. Due to its high β_2_-activity, adrenaline causes the most pronounced hyperlactatemic and hyperglycaemic responses [79, 80]. This hyperlactataemia may even be associated with short-term lactic acidosis [79, 81]. However, the extent of the catecholamines’ metabolic effects in relation to their immunomodulatory properties remains an open question [78, 82, 83]. Catecholamines contribute both directly and indirectly to stress-related insulin resistance [84] and while the catecholamine effect is already detectable under physiological conditions [45, 63], a sepsis- and/or treatment-related catecholamine desensitization due to reduced receptor density and/or affinity [85] may markedly modify this response. Noradrenaline is the first line for the management of septic shock-related arterial hypotension, and the infusion rates routinely used result in a 2- to 3-fold higher concentration than observed during physiological stress conditions [86].

**Fig. 2:**
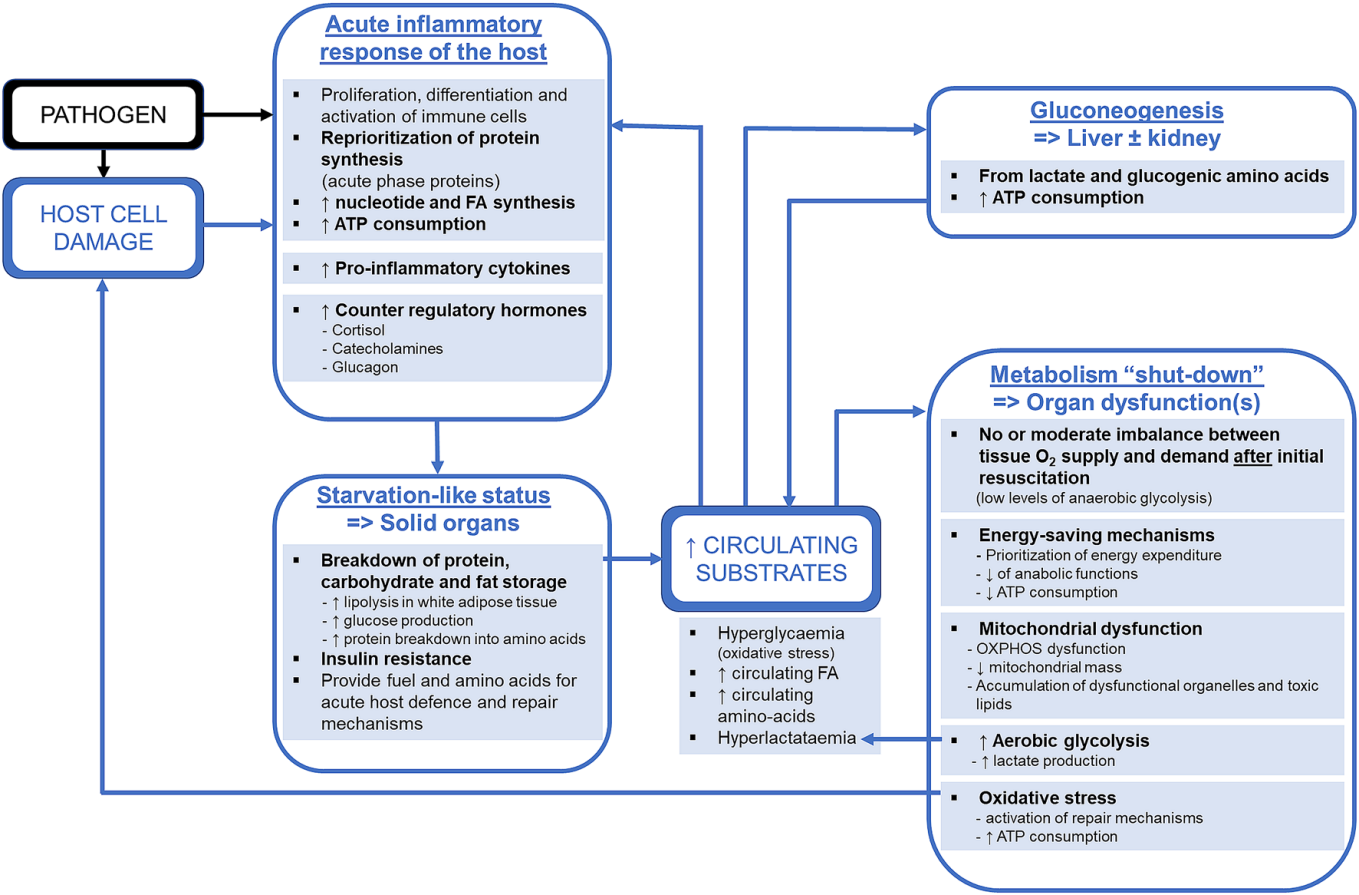
Major changes in substrate uptake and utilization in patients with sepsis. *ATP* adenosine triphosphate, *FA* fatty acids, *O*_*2*_ oxygen, *OXPHOS* oxidative phosphorylation

**Fig. 3:**
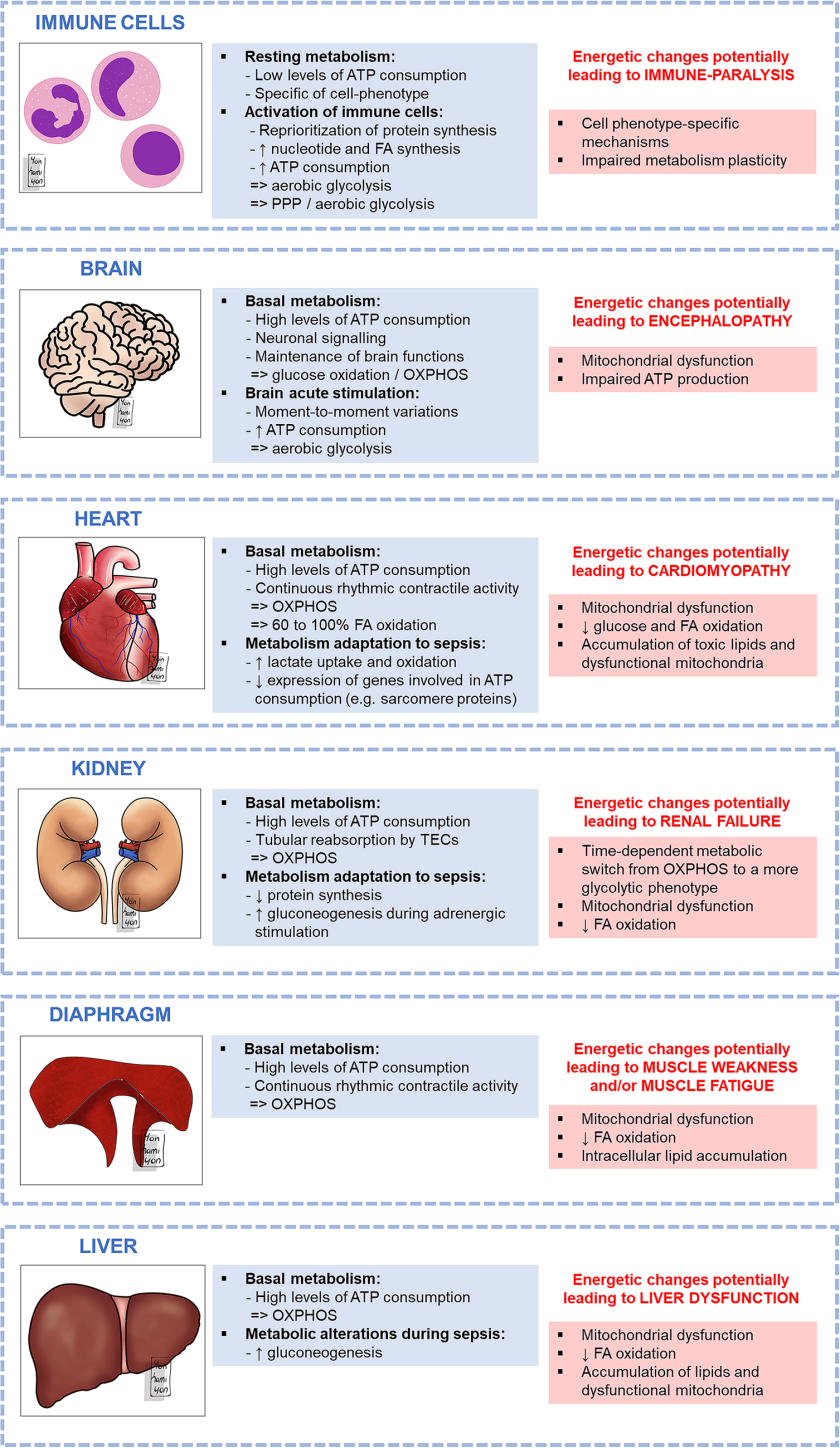
Potential sepsis-related energetic changes leading to organ dysfunction. *ATP* adenosine triphosphate, *FA* fatty acids, *OXPHOS* oxidative phosphorylation, *PPP* pentose phosphate pathway, *TEC* tubular epithelial cell

Changes in substrate uptake and utilization during sepsis or septic shock are summarized in Fig. 2. The main energetic impairments potentially responsible for sepsis-induced liver dysfunction are summarized in Fig. 3.

## Sepsis-related mitochondria dysfunction

Mitochondria generate ATP through OXPHOS and are crucial for numerous cellular functions. Far from being isolated, independent cell structures with their own DNA, mitochondria constitute a dynamic network, the quantity (biogenesis), shape and size (fusion and fission dynamics) and degradation (mitophagy) of which are tightly regulated (see the glossary in Additional file 1). Although mitochondrial impairment has been described in human sepsis for more than 18 years [23], mitochondria-targeted management remains absent from clinical practice. Moreover, many experimental models studying sepsis-related mitochondrial dysfunction involve endotoxic mice, which display an abrupt, severe cardiovascular dysfunction and a marked hypodynamic phenotype with significant falls in body temperature and metabolism [87, 88]. These models are poorly representative of the human condition, and caution should be applied in extrapolating findings in murine models to septic patients. Here, we describe the current knowledge of mitochondrial features in critically ill patients.

### Mitochondrial haplogroups

The mitochondrial DNA, localized in the matrix, encodes 13 respiratory chain subunits. Although mitochondrial DNA comprises 16,569 base pairs, its genetic background, originating from single nucleotide polymorphisms, differs among individuals and gives rise to different haplogroups. Depending on the mitochondrial DNA haplogroup, mitochondrial complex activities may differ and impact sepsis survival. In Europe, for instance, haplotypes JT and H are associated with enhanced respiratory chain activity and are associated with better survival upon admission for sepsis [[89–91]. In Asia, the mitochondrial DNA haplogroup R represents an independent factor for the outcome of sepsis in the Chinese Han population [92]. Besides haplotypes, single point mutations in mitochondrial genes can lead to increased production of reactive O_2_ species (ROS) by lymphocytes and apoptosis upon endotoxin stimulation, reinforcing the idea of a genetic component in mitochondrial susceptibility to sepsis [93].

### Mitochondrial morphology

Mitochondria are distinctive double-membrane structures with an outer membrane delimiting the organelle from the cytosol, and an involuted inner membrane forming cristae that contain respiratory complex chain proteins. Their very specific ultrastructure has been evaluated by electron microscopy in different tissues from septic patients, although caution must be used in interpreting these observations since samples were often obtained from post-mortem patients. Overall, depending on the organ (skeletal muscle, heart, liver, kidneys), biopsies from septic patient displayed only limited alterations of mitochondrial morphology, including herniation of the outer membrane, vacuoles, granules and oedematous matrix [26, 40, 94–96].

### Mitochondrial respiration and electron transport chain

Reductions in both expression and activity of complex I, III and IV have been reported in muscle obtained from critically ill subjects [97–99], and may partly explain the muscle fatigue observed in ICU patients. However, it remains equivocal as to whether altered mitochondrial bioenergetic status measured within 24 h of ICU admission is causal—i.e. responsible for the patient outcome—or merely an epiphenomenon [23, 100].

Circulating blood cell mitochondrial activity (easily measurable) has also been studied to establish whether the sepsis-related crisis in bioenergetics is detectable and could serve as a prognostic tool. Platelets from septic patients, or healthy platelets incubated with septic serum, both exhibit an increase in leak respiration (i.e. disconnected from ATP synthesis) and a higher respiratory capacity [101, 102]. Surprisingly, overall respiratory capacity is elevated in non-surviving septic patients relative to survivors and correlates with lactate levels [101, 102], suggesting a defective coupling between glucose metabolism and mitochondrial function. Conversely, cytochrome c oxidase (complex IV) activity, which decreases during sepsis [103, 104], remains higher in surviving septic patients compared to non-survivors (when measured within the first week of admission) [104]. Measuring mitochondrial function in platelets has also been reported to predict the clinical outcome in septic patients [104], but its use as a surrogate measure of mitochondrial function in other organs must be cautioned against [103]. Finally, immune cell metabolism is also of interest, though with the caveat of cell type-specific alterations of bioenergetics and metabolism (see below for details) [105]. For instance, peripheral blood mononuclear cells (PBMC) leak respiration is increased during sepsis [106, 107], meaning that mitochondrial respiration (i.e. mitochondrial VO_2_) may be rapidly uncoupled [107] and disconnected from ATP synthesis [106, 108]. Consistently, respiration coupled with ATP synthesis is lowest among non-surviving septic patients [109]. Other studies indicate no mitochondrial respiratory dysfunction in PBMC [110] or even show an increase in citrate synthase-normalized activities of complex I and IV in septic monocytes [111]. In this last study, since citrate synthase activity in monocytes from septic patients was significantly lower than in controls, a relative increase in the activity of complexes I and IV may have been magnified.

Altogether, these results highlight the tissue specificity of mitochondrial dysfunction, which can also vary according to the time of the measurement and the severity of the patient’s symptoms. Nevertheless, obtained ex vivo, the data may not precisely reflect what happens in vivo due to changes in temperature, O_2_ concentration, cytokine environment, NO concentration or light-induced nitrosylated bonds that may also change complex activities. Results may also vary according to the parameters used for normalization (i.e. citrate synthase activity, protein concentration, amount of mitochondrial DNA). Identification of other factors controlling the mitochondrial population, as well as the appropriate use of adequate experimental models, could both improve our understanding of the importance of mitochondria during sepsis.

### Mitochondrial biogenesis

Mitochondrial biogenesis arises from multiple cellular mechanisms involving several transcription factors (e.g. PPAR-γ coactivator-1α (PGC-1α), nuclear respiratory factor 1 (NRF1) and mitochondrial transcription factor A (TFAM)) which lead to new mitochondrial component synthesis and assembly, including respiratory chain subunits, antioxidant enzymes and the DNA polymerase γ.

In post-mortem biopsies, partial mitochondrial biogenesis is present in the liver and the *rectus abdominis* skeletal muscle [112] from critically ill patients *vs*. controls. Indeed, while TFAM expression and respiratory complex I activity was greater in cases’ liver and muscle than controls, DNA polymerase γ was only more expressed in the liver while mitochondrial DNA was not greater in either liver or muscle. Moreover, results may be influenced by the muscle selected, since no differences in those biogenesis markers were detected in *vastus lateralis* biopsies from the same study [112]. A decrease in mitochondrial content was also reported for intercostal and leg muscles of critically ill patients with sepsis-induced multiple organ failure [97]. Interestingly, despite no increase in respiratory complex expression or activity, the major transcription factor for mitochondrial biogenesis, PGC-1α, was 2.5-fold more expressed in *vastus lateralis* from critically ill survivors compared with controls [95], whereas PGC-1α transcription remained unchanged in non-survivors [95]. These results suggest that biogenesis activation may participate in the recovery phase.

A less invasive way to explore mitochondrial biogenesis in critical illness is available through the study of peripheral PBMC, and these studies tend to support data obtained in muscle biopsies. On admission, PGC-1α, NRF1 and TFAM messenger ribonucleic acids (RNA) from ICU patients’ PBMC were lower compared with controls, but their expression increases and remains elevated at days 3 and 5 [113], pointing to a restorative response to sepsis-induced mitochondrial depletion. The amount of mitochondrial DNA in these septic patients is consistently higher at day 5 than on admission [113], while patients with higher PGC-1α expression left the ICU after one week [113]. In line with these findings a multicentre, genome-wide expression database of 180 children ≤ 10 years old presenting with septic shock and 53 healthy controls, reported that cases with a high number of organ dysfunction were also those with the most downregulated expression of the mitochondrial, nuclear-encoded, respiratory chain complex gene in blood cells [114]. These data point to a compromised mitochondrial biogenesis in the more severe patients and, overall, activation of the biogenesis pathway may therefore represent a key prognosis factor in critically ill patients.

### Mitochondrial fission and fusion

The mitochondrial network is governed by a dynamic fusion and fission process that maintains a healthy mitochondrial population [115] by ensuring mitochondrial DNA exchange, mitochondrial DNA integrity, and also controls the size, number, distribution and maintenance of OXPHOS capacity [116].

Mitochondrial fusion is the process of by which mitochondria merge their membranes, and is controlled mainly by mitofusins 1 and 2 (MFN1/MFN2) as well as the optic atrophy 1 (OPA1) protein. On the other hand, mitochondrial fission is the process by which mitochondria divide, a process controlled mainly by dynamin-related protein 1 (DRP1) and the fission 1 (FIS1) protein.

Very few studies have explored mitochondrial dynamics in septic patients, and data may differ according to the tissue. For instance, mitofusins, OPA1 and DRP1 proteins, but not FIS1, are more expressed in post-mortem liver tissue from critically ill patients who received conventional insulin therapy than controls [112]. We could therefore speculate that the balance among septic patients may be in favour of mitochondrial fusion, since expression of the FIS1 protein apparently remains unchanged. Increased fusion could be a cellular response to improve mitochondrial function, reduce oxidative stress [117] and compensate sepsis-induced mitochondrial dysfunction. However, it may also impair the cellular process of removing damaged mitochondria (see below).

As for the biogenesis program, results differ between muscles: MFN1, MFN2, OPA1, DRP1 and FIS1 were all upregulated in *rectus abdominis* skeletal muscle biopsies from critically ill patients, while their expression was comparable to controls in the *vastus lateralis* [112]. No changes in either mitochondrial fusion or fission events in PBMC were detected between septic patients and controls [118].

Therefore, whether the balance between fusion and fission is modified by sepsis, and whether it impacts mitochondrial function and patient survival, is far from fully understood. Further studies are required to decipher the role of mitochondrial dynamics in humans with sepsis.

### Mitophagy

Dysfunctional mitochondria can be degraded by a mitochondria-targeted process called mitophagy, which shares features with the untargeted macro-autophagy. Macro-autophagy markers in the liver and skeletal muscle are more expressed in critically ill patients compared with controls, likely due to insufficient autophagic flux [119]. While the mitophagy-related proteins BCL2/adenovirus E1B 19 kDa protein-interacting protein 3 (BNIP3) and Parkin are unaltered [119], the global reduction in the autophagic flux may contribute to the accumulation of dysfunctional organelles, and hence organ failure. Autophagy activation seems to be different in renal and cardiac biopsies between septic patients and controls [26]. Although data in humans are lacking, animal and in vitro models point to an important role of auto- and mitophagy in sepsis-induced organ dysfunction, which can have either protective or deleterious effects depending on the organ [120].

### Cellular consequences of mitochondrial alterations

#### Oxidative stress

Under physiological conditions, mitochondria consume ~ 90% of the cellular O_2_, but 1–4% of the respiratory chain reactions lead to a leak of electrons that directly react with O_2_ to form O_2_^•−^, which can oxidize lipids, DNA or proteins. During sepsis, as described above, many mitochondrial-related features are altered, leading to increased generation of reactive oxygen species (ROS) [121]. This pro-inflammatory environment also triggers inducible NO synthase (NOS)-dependent NO production which, in turn, may react with O_2_^•−^to form the highly reactive ONOO− that has been shown to impair mitochondrial function [122]. Enzymatic defences such as the mitochondrial superoxide dismutase 2 (SOD2, also known as Mn-SOD) converts O_2_^•−^ into hydrogen peroxide (H2O2), which can then be detoxified into water by the catalase or the selenium-containing glutathione peroxidase. SOD2 expression, as for PGC-1α, is more expressed in survivors of critical illness [95], suggesting a protective role of this detoxifying enzyme. However, higher SOD activity observed in septic patients with the SOD2 gene 47C > T single nucleotide polymorphism [123] increases the risks of developing septic shock. The authors suggest that the excess of SOD2 activity may increase the production of H2O2, which would amplify mitochondrial dysfunction. Glutathione and CoQ10 (known as the lipid-soluble ubiquinone, carrying electrons from complex I and II to complex III) are also important antioxidants localized in mitochondria. Studies indicate that CoQ10 levels are significantly lower in septic shock patients than in healthy controls, which could contribute to mitochondrial dysfunction [58]. However, initial studies on oral CoQ10 administration did not report modified amounts of cytochrome c, mitochondrial DNA, mitochondrial RNA or inflammatory parameters [59]. But these results have recently been challenged, administration of 100 mg CoQ10 twice a day for seven days, added to standard sepsis treatment, having been shown to reduce inflammation, oxidative stress and mortality, and improve mitochondrial function [124].

#### Cell death

Mitochondria may also be viewed as a signalling platform regulating cell death [125]. Upon mitochondrial stress (e.g. oxidative stress, calcium overload), they can release pro-apoptotic factors such as cytochrome c. Interestingly, total B and T lymphocytes are reduced in critically ill patients: these cells undergo apoptosis [126, 127]. Importantly, caspase-9 activation has been detected, suggesting the involvement of mitochondria [126]. Moreover, patients with sepsis or septic shock exhibit lower levels of the mitochondrial anti-apoptotic protein B-cell lymphoma 2 (BCL2) in their lymphocytes [127]. Mitochondrial depolarization, which occurs during apoptosis, is also detected in septic monocytes [128, 129]. A positive correlation has been found between the number of cells with depolarized mitochondria and mortality [129]. Moreover, sepsis survivors exhibited higher BCL2 levels [129]. Mitochondrial-dependent apoptosis may therefore contribute to the sepsis-associated immune-paralysis and in-hospital mortality.

**Fig. 4:**
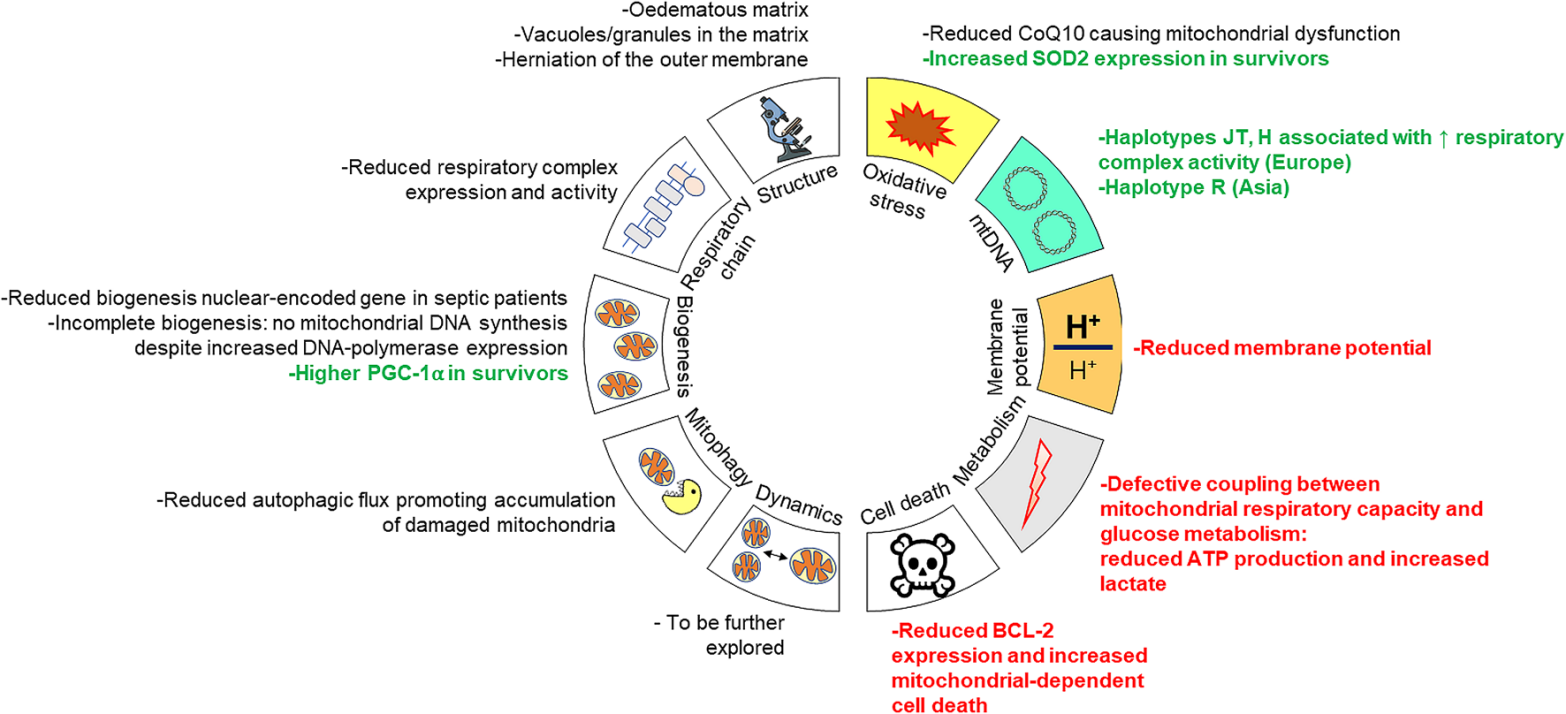
Hallmarks of mitochondrial changes observed during human sepsis and septic shock. Bold red and green fonts refer to the association with increased mortality or survival, respectively. In the other cases, no correlations have been made. *ATP* adenosine triphosphate, *BCL2* B-cell lymphoma-2, *CoQ10* coenzyme Q10, *DNA* desoxyribonucleic acid, *PGC-1α* PPAR-γ coactivator-1α, *SOD2* superoxide dismutase 2

The hallmarks of mitochondrial changes observed during human sepsis or septic shock are summarized in Fig. 4.

## Immuno-metabolism

During inflammation, immune cell responses are highly dynamic and require continuous metabolic adaption to maintain a sufficient host defence. The bioenergetic demands are usually met by the interconnected metabolic pathways of glycolysis, the tricarboxylic acid (TCA) cycle and OXPHOS. Other sources, such as FA and glutamine, can fuel the TCA cycle. Some types of immune cell preferentially use aerobic glycolysis for ATP production (similar to the so-called Warburg effect in cancer cells, where glucose is converted to pyruvate and then diverted to lactate rather than entering the Krebs' cycle with carbon dioxide (CO_2_) production in the presence of sufficient amounts of O_2_ [130]).

**Fig. 5:**
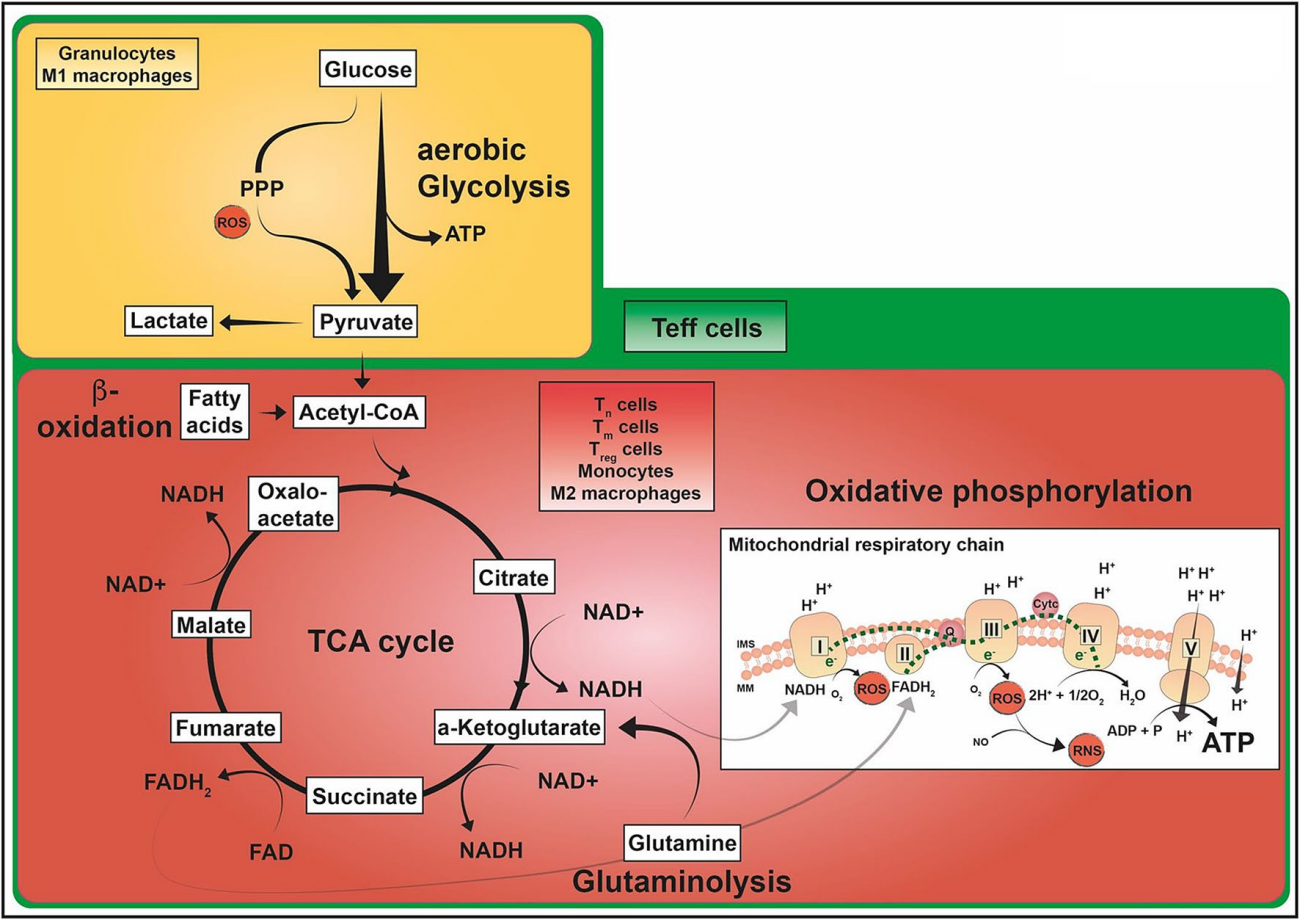
ATP-producing metabolic pathways in distinct immune cell subsets. Glucose oxidation to pyruvate via glycolysis is a fast reaction generating 2 mol of ATP per mol glucose. This aerobic glycolysis is complemented by the PPP that can produce further metabolic precursor molecules and is involved in ROS production. Pyruvate can be converted to lactate or further oxidized to acetyl-CoA entering the mitochondrial TCA cycle (yellow box). The TCA cycle (red box) generates reducing equivalents NADH and FADH2 which are utilized in the mitochondrial respiratory chain to build up the proton gradient across the mitochondrial inner membrane by complexes I–IV of the respiratory chain. As a by-product, ROS and RNS are produced. OXPHOS produces larger amounts of ATP (~ 30 mol/mol glucose) by complex V. Immune cells are also able to utilize substrates such as glutamine, which enters these pathways via the TCA metabolite α-ketoglutarate and FA, which are oxidized to acetyl-CoA via β-oxidation. Granulocytes and M1 macrophages (yellow box) have a highly glycolytic metabolism even when O_2_ is available. Their TCA cycle and respiratory chain activity are maintained at a low level. T_n_, T_m_, and T_reg_ cells, as well as monocytes and M2 macrophages, primarily perform OXPHOS and are also able to metabolize FA and glutamine in order to fuel the TCA cycle (red box). T_eff_ cells (green box) have a highly active metabolism including all of the pathways described. *ADP* adenosine diphosphate, *ATP* adenosine triphosphate, *CoA* coenzyme A, *FADH*_*2*_*/FAD* flavin adenine dinucleotide in its reduced/oxidized form, *H*_*2*_*O* water, *IMS* intermembrane space, *MM* mitochondrial membrane, NADH/NAD^+^ nicotinamide adenine dinucleotide in its reduced/oxidized form, *O*_*2*_ oxygen, *OXPHOS* oxidative phosphorylation, *PPP* pentose phosphate pathway, *ROS* reactive O_2_ species, *RNS* reactive nitrogen species, *TCA* tricarboxylic acid cycle, *T*_*eff*_ effector T cell, *T*_*n*_ naïve T cell, *T*_*m*_ memory T cell, *T*_*reg*_ regulatory T cell. Reprinted with permission from Zhang et al. 2020 [105]

Aerobic glycolysis (2 mol ATP/mol glucose) is far less efficient than complete glucose oxidation via glycolysis and the subsequent TCA cycle and OXPHOS (theoretical maximum ≈ 30–33 mol ATP/ mol glucose [131]. This difference can be at least partly offset by the rapidity of aerobic glycolysis [132]. High-throughput aerobic glycolysis subsequently increases the flux of glucose 6-phosphate through the pentose phosphate pathway (PPP), a metabolic shunt that parallels glycolysis. The PPP generates five-carbon sugars for nucleotide synthesis and reduced nicotinamide adenine dinucleotide phosphate (NADPH), crucial for the NADPH-dependent respiratory burst and FA biosynthesis (Fig. 5, reproduced with permission from Zhang et al. [105]).

The high plasticity of immune cell metabolism is crucial to their ability to fulfil different changing functions, and, consequently, loss of this plasticity has been referred to as a potential reason for sepsis-associated immune-paralysis [133, 134].

### Granulocytes

Neutrophils have only a few mitochondria, and consequently, at rest, consume only small amounts of O_2_. Upon activation—that is, with initiation of chemotaxis and phagocytosis [135]—ATP turnover remains mostly unaffected [136, 137], whereas O_2_ requirements markedly increase due to the respiratory burst. Glycolysis is crucial for phagocytosis and the formation of neutrophil extracellular traps (NETs) [65, 138]. The high flux through the glycolytic pathway increases the flux through the PPP, in order to generate NADPH, which is then used by the NADPH oxidase (NOX-2) [139] to generate O_2_^• –^, which is converted to H_2_O_2_ mainly via its reaction with SOD [140]. This mechanism is widely referred to as the “oxidative burst” [140].

### Monocytes

Activated monocytes eliminate pathogens via phagocytosis, ROS and cytokine production (among other mechanisms), by shifting their metabolism to a more glycolytic phenotype (Fig. 5, reproduced with permission from Zhang et al. [105]), though “maintenance functions” remain dependent upon OXPHOS activation-related processes. In addition, monocytes are recruited to inflammatory tissue and continue to differentiate into two subtypes: M1 macrophages, promoting a pro-inflammatory response; or else the anti-inflammatory M2 macrophages, which play a crucial role in inflammation resolution and tissue healing. The pro-inflammatory M1 subtype relies upon aerobic glycolysis, and hence exhibits low VO_2_ rates. During the resolution of inflammation, macrophages differentiate into the M2 phenotype, which primarily utilises OXPHOS metabolism fuelled by FA oxidation. In other words, the two functional macrophage states (M1 *vs.* M2) correspond to a time-dependent sequence of metabolic activity, with enhanced glycolysis and PPP turnover during the activation phase, and a return to TCA and OXPHOS during the resolution phase of inflammation [141, 142].

### T-lymphocytes

Unstimulated, naïve T lymphocytes primarily use OXPHOS to generate ATP, and their function is related to their metabolic activity [143]. Activated T-lymphocytes rapidly proliferate and differentiate into subpopulations, such as effector T cells (T_eff_), regulatory T cells (T_reg_), and memory T cells (T_m_). The T_eff_ cells have a low mitochondrial mass, a correspondingly low spare respiratory capacity, and generate ATP predominantly through aerobic glycolysis; in contrast, T_reg_ cells and T_m_ cells depend mainly on OXPHOS and FA oxidation for ATP generation. The T_reg_ cells use oxidative metabolism, but also exhibit aerobic glycolysis, while T_m_ cells embody features of both naïve and effector cells. Finally, T_m_ cells have more mitochondrial mass and a higher spare respiratory capacity than naïve cells.

### B-lymphocytes

While data on B-cell lymphocyte metabolism are scarce, recent evidence indicates that resting B-cells have lower energy requirements than resting T-cells as they consume less glucose and FA and, consequently, produce less ATP [144]. Nevertheless, they rely primarily on OXPHOS to meet their metabolic demands and have a higher mitochondrial mass. Despite these differences in the resting state, B cells share some metabolic characteristics with T cells upon activation.

The main energetic impairments potentially responsible for sepsis-induced immune-paralysis are summarized in Fig. 3.

## Sepsis-related encephalopathy

The brain’s energy demands are remarkable, both in intensity and in their dynamic range from moment-to-moment. It is well established that the brain consumes a disproportionate amount of ATP compared to other organs, estimated at as much as 20% of total ATP consumption for an organ that represents only 2% of the body’s mass [145]. This makes sense: the electrochemical reactions that drive neuronal signalling are energetically expensive. Neurons maintain a hyperpolarized resting membrane potential, ship proteins throughout an expansive cellular architecture, maintain an enormous membrane surface area, and continuously release and recycle synaptic vesicles at each of the trillions of synapses throughout the brain. In addition, there is an enormous dynamic variation in the ATP consumption of brain cells from moment to moment as the brain engages in its signalling.

### Fuelling thought: mechanisms of brain energy homeostasis

Despite the importance of metabolic signalling for the maintenance of brain function, our understanding of how the metabolic pathways responsible for the homeostatic maintenance of cellular ATP is limited. Unlike other tissues, there is only a tiny metabolization of long-chain FA in the brain, which probably minimizes peroxidation of polyunsaturated long-chain FA. Nevertheless, at least under conditions of limited O_2_ supply, neurons are capable of oxidizing ketone bodies as well as odd-numbered, medium-chain FA, the latter to maintain citric cycle intermediate levels [146, 147]. Furthermore, unlike muscles, the brain does not maintain appreciable fuel stocks in the form of glycogen. Although astrocytes maintain a glycogen reserve, resting neurons rely primarily on fuel delivered in the form of blood glucose, with a near-stoichiometric utilization of O_2_ and glucose [148]. The coupling of cerebral blood flow and metabolism is the main strength of this well-tuned energy supply machinery. During neural activation, an increase in neuronal activity is associated with increased blood flow, providing both O_2_ and glucose and eliminating CO_2_. Nevertheless, the dynamic control of blood supply does not explain the entire story and the energy demands of neurons throughout the brain change constantly on a cell-by-cell basis. Interestingly, evidence is accumulating that neurons must synthesize ATP as required, responding to continually changing demands of fluctuating neural activity [149]. To do so, neurons seem to take advantage of both glycolysis and OXPHOS to produce ATP from circulating glucose. In fact, aerobic glycolysis occurs during the transient metabolic response of the brain to acute stimulation [149]. Overall, like fast-twitch muscle fibres, the brain may temporarily allow glycolysis to exceed OXPHOS in the face of acute energy demand.

### Septic encephalopathy: metabolic underpinnings

In sepsis, cerebral metabolism is radically altered and experimental evidence has shown that oxidative stress occurs in in the brain in the early stages of sepsis, and that this results in decreased ATP synthesis [150]. Oxidative stress and the enhanced production of cytokines (interleukin-1β and interleukin-2) seem to have neurotoxic effects on the brain, mediated by by-products of the interaction between free electrons and O_2_ production such as O_2_^• –^, H_2_O_2_, or hydroxyl radicals. Although only a small amount of O_2_ consumed is converted to ROS, these short-lived and highly reactive molecules can inflict damage on the mitochondrial electron transport chain and destroy mitochondrial structural components. For example, studies have shown increased brain SOD-to-catalase activity, leading to increased H_2_O_2_ production in rats during early sepsis [151]. In fact, inhibition of mitochondrial function during sepsis may be a significant cause of impaired O_2_ utilization and metabolism in the brain, probably driven by NO-mediated mechanisms [151]. Bacterial toxins directly induce activation of cerebral microglia by increased expression of cell-specific targeted antigen, leading to amplified production of NO and provoking significant dysfunction in redox signalling [152]. Further studies are needed to explore the potential therapeutic use of this mechanistic knowledge in patients.

### Sepsis, ATP and higher brain functions

Sepsis-induced encephalopathy is clinically characterized by an alteration of consciousness, which ranges from confusion (i.e. *delirium*) to coma and is associated with increased mortality and long-term cognitive dysfunction [150, 152, 153]. The pathophysiology of sepsis-induced, multiple organ dysfunction is not fully understood [154]. However, existing data indicate an important role for inflammatory processes and diffuse endothelial activations with microcirculatory and blood–brain barrier dysfunction, triggered by the release of inflammatory mediators. In animal models and septic patients, microglial activation has been reported in multiple neuropathological studies [154]. Neurotoxic and neural apoptosis processes occurring during sepsis have been documented by converging neuroanatomical studies [152, 154]. Interestingly, long-term cognitive impairment in sepsis survivors seems to be related to brain mitochondrial dysfunction, and activators of autophagy, mitophagy and mitochondrial biogenesis rescue septic animals from cognitive impairment [155]. Ultimately, an understanding of the connections between basic metabolic biochemistry and higher brain functions is likely to significantly impact the clinical management of sepsis-associated encephalopathy.

The main energetic impairments potentially responsible for sepsis-induced encephalopathy are summarized in Fig. 3.

## Septic cardiomyopathy

Growing evidence associates septic cardiac dysfunction with impaired metabolism and limited energy production leading to changes in substrates consumption and accumulation of toxic lipids in cardiomyocytes.

The normal heart produces approximately 60–100% of its energy from free FA and the remainder from glucose and lactate (0–20% from each) [156]. In 1987, Dhainaut et al. demonstrated that patients in septic shock undergo significant changes in myocardial substrate uptake when compared with control patients [157]: lactate uptake was increased, while free FA, glucose and ketone body uptake were all significantly decreased. Non-survivors had lower myocardial FA and glucose uptake than survivors.

Surprisingly, despite reduced cardiac lipid uptake, sepsis is associated with intracellular lipid accumulation in the myocardium of patients who died from sepsis [158]. Lipid accumulation in the heart has been attributed to an impaired FA oxidation in animal models of sepsis [159]. The decrease in both FA and glucose oxidation in the myocardium is a potential cause of reduced ATP turnover, which leads to myocardial contractile dysfunction and sometimes death [157, 159–163]. Moreover, during sepsis, the lipid accumulation (e.g. sphingosine) in cardiomyocytes may be toxic and directly provoke contractile and mitochondria dysfunction [159, 164, 165].

Prevention or reversal of sepsis-related alteration of FA oxidation may be a therapeutic target. Interventional studies on mammal models suggest that modulating PPAR’s activity may promote the transcription of β-oxidation- and mitochondrial biogenesis-related genes, and ultimately prevent mitochondria and contractile dysfunction of the heart in sepsis cases. Firstly, systemic pharmacological activation of PPAR-γ or PPAR-β/δ [159, 166, 167], and prevention of PPAR-α decrease [163], attenuated myocardial dysfunction and increased survival in murine models of sepsis. Secondly, genetic stimulation of PGC-1β expression, a transcriptional coactivator of PPARs which promotes cardiac FA oxidation and mitochondrial biogenesis, prevented contractile dysfunction of the heart in mice challenged with an endotoxin injection [168]. Of note, Standage et al. have shown that PPAR-α was necessary to induce the hyperdynamic cardiac response in the early stage of sepsis in a mouse model of caecal ligation and puncture [169].

Sepsis-related mitochondrial dysfunction in the myocardium is multifactorial, and detailed mechanisms underlying this dysfunction have been reviewed elsewhere [170–172]. Mitochondrial dysfunction in murine septic cardiomyopathy is characterized by both decreased rates of mitochondrial respiration coupled with ATP synthesis, and increased rates of leak respiration [173–176]. Respiratory chain malfunction has been associated with contractile defects, and prevention of OXPHOS impairment is known to prevent cardiac dysfunction in murine models of sepsis [173–177].

Since the mitochondrial capacity for generating ATP is decreased in sepsis, reducing the expression of genes involved in ATP consumption (i.e. sarcomeric contraction and excitation–contraction coupling) may be a protective mechanism against myocardial necrosis in human septic shock [24, 26, 178]. The importance of mitochondria in cellular signalling and homeostasis could provide a mechanistic explanation for the link between mitochondrial dysfunction and heart failure in septic cardiomyopathy [179]. Indeed, mitochondrial ROS production [180, 181], or externalization of other mitochondrial components in the cytosol (e.g. cytochrome c, SMAC/diablo, PGAM5, and mitochondrial DNA) [182–184], are involved in both caspase activation and inflammation, both important features in the pathophysiology of endotoxin-induced cardiac dysfunction [185, 186].

Once mitochondrial damage has occurred, recovery may depend upon the efficiency of removal and replacement of damaged mitochondria. In the septic cardiomyocyte, there is an early decrease in mitochondrial mass, in parallel with an early activation of biogenesis [166, 187–189] and mitochondrial autophagy [175, 190–192]. Genetic or pharmacological activation of mitochondrial biogenesis or autophagy prevented mitochondrial and contractile dysfunction in the heart of different murine models of sepsis [175, 177, 190, 191]. Moreover, Parkin E3 ubiquitin ligase, a key effector of mitophagy in mammal cells, was shown to be required for complete recovery of mitochondrial and cardiac function in endotoxic mice [193]. Nonetheless, the beneficial effects of autophagy stimulation and/or mitochondrial biogenesis activation are not always consistent and may, for example, be blunted in the heart of mature subjects [194, 195].

The main energetic impairments potentially responsible for sepsis-induced cardiomyopathy are summarized in Fig. 3.

## Sepsis-induced acute kidney injury

The kidney has one of the highest metabolic resting rates among organs of the body [196] and, after the heart, contains the second-highest number of mitochondria [197, 198]. The kidney consumes almost 7% of the body’s daily ATP expenditure [199] to perform various functions, but most of the ATP produced is used to generate ion gradients across cellular membranes to enable active reabsorption [200]. Consequently, renal tubular epithelial cells (TECs), which actively reabsorb 80% of the filtrate filtered by the glomeruli, have the highest mitochondria density and are highly impacted in the event of mitochondrial dysfunction [201].

During sepsis-induced AKI, significant metabolic adaptations occur. These include:

- **A reprioritization of ATP consumption to prevent epithelial cell death** [202]**:** a decrease in energy expenditure for non-vital functions is observed in TECs during sepsis. A translation shut-down (blockade of protein synthesis) occurs in murine endotoxaemia, and endotoxins and/or pro-inflammatory cytokines have been shown to downregulate renal transporters/channels [202–205]. The energy thus economized is used to maintain TECs’ vital functions, including membrane Na + /K + ATPase pump that has an essential role in cell life and death [202].
- **A reprograming of renal cell metabolism** [202]: during sepsis, a biphasic metabolic reprograming is observed in TECs. First, sepsis induces an early and acute pro-inflammatory phase (shift of metabolism from OXPHOS towards a more glycolytic phenotype), followed by a late catabolic anti-inflammatory response (inverse shift; restoration of predominant OXPHOS metabolism) [206]. In TECs, the first switch has been described in murine endotoxaemia [206–208], while the second remains more hypothetical and is derived from studies in actively proliferating tumour cells [206]. The temporary increase in aerobic glycolysis is surprising since it is less efficient than OXPHOS for generating ATP [209]. Hypotheses regarding the usefulness of this switch include the limitation of oxidative damage (decrease in ROS production, regeneration of glutathione), and providing the opportunity for TECs to generate anti-inflammatory response/signals while still producing enough energy to prevent cell death by supplying key components necessary for vital functions and mitosis (FA, amino acids and nucleotides) [206]. Interestingly, experimental studies in animals have shown that induction of OXPHOS/inhibition of aerobic glycolysis was associated with less AKI onset and reduced mortality [210, 211]. However, the harmfulness of aerobic glycolysis seems to be time-dependant: sirtuin-1 inhibition in mice coincident with caecal ligation and puncture increased mortality, while it improved survival when inhibited 24 h later (sirtuin-1 inhibits aerobic glycolysis and promotes β-oxidation of FA and OXPHOS) [212].
- **A restoration of the mitochondrial pool** [202]: mitochondrial dysfunction contributes to sepsis-induced AKI. In a murine model of sepsis, Tran et al. demonstrated mitochondrial swelling and disruption of cristae in renal TECs (which occurs prior to the onset of AKI), suggesting a pathogenic role of mitochondrial dysfunction in sepsis-induced AKI [213]. Mitochondrial biogenesis is also an important process that occurs during sepsis-induced AKI. A positive regulator of mitochondrial biogenesis, PGC-1α, decreased in a manner proportional to the degree of renal impairment [213]. When mice recovered from sepsis, PGC-1α returned to normal levels. Moreover, aberrant fission/fusion has been observed during sepsis-induced AKI in mice [214]. Along with mitochondrial biogenesis, mitophagy is also necessary to preserve the mitochondrial pool. Dysfunctional mitochondria can be degraded by mitophagy allowing the turnover of injured mitochondria by signalling, and engulfing them into autophagosomes for degradation. Kidney mitophagy was elevated in early stage, sepsis-induced AKI, while it was impaired in the later phase [214].

The main energetic impairments potentially responsible for sepsis-induced AKI are summarized in Fig. 3.

## Sepsis-induced diaphragmatic dysfunction

The diaphragm has unique properties compared with other striated muscles:

- It contracts with a continuous rhythmic activity through the life span.
- Its resting position and length change with numerous variables such as body position, elastic recoil of the lungs, gravity effects on the chest wall, and content of the abdomen and the thorax.
- Diaphragm myofibres have a higher oxidative capacity, higher capillary density, higher maximal blood flow and resistance to fatigue than limb muscles [215].

Its higher susceptibility to inflammation [216] and metabolic stress [217] may predispose the diaphragm to the deleterious consequences of sepsis, namely atrophy and loss of force [218, 219]. In a seminal study, Hussain et al. showed that endotoxaemic shock was associated with hypercapnic respiratory failure [220]. Subsequent studies explored the different mechanisms of sepsis-induced diaphragmatic function and reported that several pathways are involved: oxidative stress, inflammation, altered proteostasis and metabolic/mitochondrial dysfunction [221]. Sepsis-induced metabolic/mitochondrial dysfunction can be summarized by several key findings. Sepsis is associated with a reduction of transcripts and protein levels for mitochondrial electron transport chain components and phosphofructokinase (the rate-limiting glycolysis enzyme), as well as a decrease in the rates of mitochondrial respiration (coupled with ATP synthesis) and phosphofructokinase enzyme activity [222]. Along with the reduction in mitochondrial function as the energy provider to the cell, endotoxemic shock is associated with excessive production of ROS [223]. In septic rats, a burst in NO formation was associated with increased intra-mitochondrial O_2_^•—^concentrations and protein nitrosylations. The production of ROS was associated with a loss in production of diaphragm force, which, in turn, was prevented by the administration of a NOS inhibitor. Few studies have been performed in humans. Van den Berg et al. collected diaphragm biopsies performed during abdominal surgery in 36 critically ill patients, 58% of whom presented ongoing sepsis [224]. Biopsies were performed when surgery was needed during the ICU stay. The authors reported conserved mitochondrial respiration, energy status and morphology despite atrophy and reduced force. Nevertheless, mitochondrial fusion proteins (mitofusins and OPA1) and PGC1-⍺, which promote mitochondrial biogenesis, were decreased in critically ill patients compared with controls.

Sepsis and mechanical ventilation are both associated with lipid accumulation in the diaphragm [217, 225]. In mechanically ventilated, brain-dead organ donors, optical microscopy with oil red O staining showed lipid droplets in the diaphragm along with elevated transcripts for FA synthase and adipokines. The gene expression of a FA transporter to mitochondria, carnitine palmitoyl transferase-1, was also downregulated. The imbalance between an increased level of the metabolic sensor adenosine monophosphate-activated protein kinase (AMPK) and the decreased level of sirtuin-3, a mitochondrial sirtuin implicated in mitochondrial biogenesis and FA oxidation, was held to favour diaphragmatic metabolic oversupply [217]. This profile was reproduced in endotoxemic rabbits [225]. Along with consequences for lipid metabolism, glucose metabolism is also impaired during sepsis. In limb muscle biopsies from patients with ICU-acquired weakness, the insulin-dependent glucose transporter from plasma to adipocytes and muscle cells, GLUT4, was trapped in perinuclear spaces instead of being located at its usual location at the sarcolemma. This mis-translocation of GLUT4 was associated with insulin resistance and was reversed by electrical muscle stimulation [226], but whether GLUT4 mis-location also exists in the respiratory muscles with functional consequences is hypothetical.

Although there are numerous possibilities to treat sepsis-induced diaphragmatic dysfunction by targeting mitochondria, none have as yet been proven to improve prognosis. These opportunities may include mitochondrial antioxidants [227–229], drugs such as sirtuin activators (e.g. resveratrol) [230] or bezafibrate to promote mitochondrial biogenesis [231] (which is associated with survival in critically ill septic patients [95]), or even mitochondrial transplantation [232]. However, despite the abundant evidence for increased oxidative stress in patients with sepsis, treatment with antioxidants may have major potential pitfalls: (1) oxidative damage is related to the tissue rather than the blood antioxidant capacity, which may itself be inversely related to the severity of organ failure [233, 234]. This is, at least in part, due to bilirubin, a potent endogenous antioxidant but also one of the markers of liver dysfunction/failure; (2) clearly, depending on the surrounding “milieu” any antioxidant (at least of non-enzymatic origin) can become a pro-oxidant and thus eventually even cause harm, a phenomenon referred to as the “antioxidant paradox” [235, 236], with albumin being a prominent example [237, 238]; (3) both ROS and reactive nitrogen species (RNS) assume crucial importance, both as signalling molecules and for antimicrobial host defence, so that their complete eradication will most likely have deleterious consequences [239]. It is therefore not surprising that—except for the controversial combination of hydrocortisone, vitamin c and thiamine [221, 222, 240]—antioxidant interventions have so far failed to improve outcomes [223]. Finally, treatment may also include the promotion of spontaneous breathing, which was reported to enhance mitochondrial respiration [241], decrease ROS generation, optimize substrate utilization, avoid metabolic oversupply and prevent loss of diaphragmatic force [242, 243].

The main energetic impairments potentially responsible for sepsis-induced diaphragmatic dysfunction are summarized in Fig. 3.

## Conclusion

Systemic and organ-specific changes in bioenergetics and metabolism characterize the acute phase of sepsis or septic shock. Alterations of VO_2_, increased levels of circulating substrates, impaired glucose and lipid oxidation, and mitochondrial dysfunction are all associated with organ dysfunction and poor outcomes during ICU stay. Recent understandings in substrate utilization and mitochondrial dysfunction may pave the way for new diagnostic and therapeutic approaches. These findings could help physicians to identify distinct subgroups of sepsis and, subsequently, to personalize treatment strategies. Implications for their use as bioenergetic targets to identify metabolism- and mitochondria-targeted treatments need to be evaluated in future studies.

## Supplementary Information

**Additional file 1.** Glossary of key terms in energetic metabolism

## Data Availability

Not applicable.

## Abbreviations

AMPK: Adenosine monophosphate-activated protein kinase
AKI: Acute kidney injury
ANLS: Astrocyte-to-neuron lactate shuttle
ATP: Adenosine triphosphate
BCL2: B-cell lymphoma 2 protein
BNIP3: BCL2/adenovirus E1B 19 kDa protein-interacting protein 3
CO_2_: Carbon dioxide
CoQ10: Coenzyme Q10
DO_2_: O_2_delivery
DNA: Desoxyribonucleic acid
DRP1: Dynamin-related protein 1
FA: Fatty acids
FADH2: Reduced flavin adenine dinucleotide
FIS1: Fission 1
H_2_O: Water
H_2_O_2_: Hydrogen peroxide
ICU: Intensive care unit
MFN1 and MFN2: Mitofusins 1 and 2, respectively
NADH: Reduced nicotinamide adenine dinucleotide
NADPH: Reduced nicotinamide adenine dinucleotide phosphate
NETs: Neutrophil extracellular traps
NO: Nitric oxide
NOS: NO synthase
NOX: NADPH oxidase
NRF: Nuclear respiratory factor
O_2_: Oxygen
O_2_^• −^: Superoxide anion
ONOO^−^: Peroxynitrite
OPA1: Pptic atrophy 1
OXPHOX: Oxidative phosphorylation
PBMC: Peripheral blood mononuclear cells
PGC-1: PPAR-γ coactivator-1
PPAR: Peroxisome proliferator-activated receptor
PPP: Pentose phosphate pathway
SOD: Superoxide dismutase
RNA: Rribonucleic acid
RNS: Reactive nitrogen species
ROS: Reactive O_2_ species
TCA cycle: Tricarboxylic acid cycle
T_eff_: Effector T lymphocyte
TEC: Tubular epithelial cell
TFAM: Mitochondrial transcription factor A
T_m_: Memory T lymphocyte
T_n_: Naive T lymphocyte
T_reg_: Regulatory T lymphocyte
VO_2_: O_2_ Consumption
