# Correction to: Energetic dysfunction in sepsis: a narrative review

**DOI:** 10.1186/s13613-021-00970-x

**Published:** 2021-12-29

**Authors:** Sebastien Preau, Dominique Vodovar, Boris Jung, Steve Lancel, Lara Zafrani, Aurelien Flatres, Mehdi Oualha, Guillaume Voiriot, Youenn Jouan, Jeremie Joffre, Fabrice Uhel, Nicolas De Prost, Stein Silva, Eric Azabou, Peter Radermacher

**Affiliations:** 1 U1167 - RID-AGE - Facteurs de Risque Et Déterminants Moléculaires Des Maladies Liées Au Vieillissement, University Lille, Inserm, CHU Lille, Institut Pasteur de Lille, 59000 Lille, France; 2 Centre AntiPoison de Paris, Hôpital Fernand Widal, APHP, 75010 Paris, France; 3 Faculté de Pharmacie, UMRS 1144, 75006 Paris, France; 4 Université de Paris, UFR de Médecine, 75010 Paris, France; 5 Medical Intensive Care Unit, Lapeyronie Teaching Hospital, Montpellier University Hospital and PhyMedExp, University of Montpellier, Montpellier, France; 6 Médecine Intensive Réanimation, Hôpital Saint-Louis, AP-HP, Université de Paris, Paris, France; 7 INSERMUMR 976,Hôpital Saint Louis, Université de Paris, Paris, France; 8 PhyMedExp, University of Montpellier, Montpellier, France; 9 Pediatric Intensive Care Unit, Necker Hospital, APHP, Centre - Paris University, Paris, France; 10 Service de Médecine Intensive Réanimation, Sorbonne Université, Assistance Publique - Hôpitaux de Paris, Hôpital Tenon, Paris, France; 11 Service de Médecine Intensive Réanimation, CHRU Tours, Tours, France; 12 Faculté de Médecine de Tours, INSERM U1100 Centre d’Etudes Des Pathologies Respiratoires, Tours, France; 13 Department of Anesthesia and Perioperative Care, University of California, San Francisco, CA 94143 USA; 14 Réanimation Médico-ChirurgicaleUniversité de Paris, Assistance Publique - Hôpitaux de Paris, Hôpital Louis Mourier, Paris, France; 15 Service de Réanimation MédicaleHôpital Henri Mondor, Assistance Publique-Hôpitaux de Paris, 94010 Créteil, France; 16 Réanimation URM CHU Purpan, Cedex 31300 Toulouse, France; 17 Toulouse NeuroImaging Center INSERM1214, Cedex 31300 Toulouse, France; 18 Clinical Neurophysiology and Neuromodulation Unit, Departments of Physiology and Critical Care Medicine, Raymond Poincaré Hospital, AP-HP, Inserm UMR 1173, Infection and Infammation (2I), University of Versailles (UVSQ), Paris-Saclay University, Paris, France; 19 Institut Für Anästhesiologische Pathophysiologie Und VerfahrensentwicklungUniversitätsklinikum, Ulm, Germany

## Correction to: Ann Intensive Care (2021) 11:104 https://doi.org/10.1186/s13613-021-00893-7

In the original publication of the article [1], the eleventh author’s name was misspelt as Fabrice Huel instead of Fabrice Uhel. The original article has been corrected.
