# Supplementary material for: Energetic dysfunction in sepsis: a narrative review
Source: Ann Intensive Care. 2021 Jul 3;11:104. doi: 10.1186/s13613-021-00893-7 (PMC8254847; doi:10.1186/s13613-021-00893-7)
Supplement: Supplementary file 1 — Additional file 1. Glossary of key terms in energetic metabolism [file 13613_2021_893_MOESM1_ESM.docx]

**Additional file 1**. Glossary of key terms in energetic metabolism

| ***Aerobic glycolysis***  ***-***  ***Warburg-like aerobic glycolysis*** | In the presence of O2 along with a high glycolysis to OXPHOS activity ratio, a part of glycolysis is not followed by OXPHOS (i.e. Warburg-like aerobic glycolysis or aerobic glycolysis). Certain cell subsets (e.g. immune cells upon activation) preferentially use aerobic glycolysis for ATP production, a metabolic status first described in cancer and referred to as Warburg effect [1].  Aerobic glycolysis only (2 mol ATP/ mol glucose) is far less efficient to generate ATP compared to aerobic glycolysis that is followed by OXPHOS pathways (≈ 30-33 mol ATP/ mol glucose). |
| --- | --- |
| ***Anaerobic glycolysis*** | Under anoxic or hypoxic conditions, ATP is generated via anaerobic glycolysis by converting pyruvate to lactate.  Anaerobic glycolysis (2 mol ATP/ mol glucose) is far less efficient to generate ATP compared to aerobic glycolysis that is followed by OXPHOS pathways (≈30-33 mol ATP/ mol glucose). |
| ***β-oxidation*** | The metabolic pathway in which FA are converted to acetyl-CoA. Thus, FA derived from both the plasma and the breakdown of intracellular triacylglycerol stores, can fuel the TCA cycle. In all mammals, β-oxidation takes place inside mitochondria.  Activations of PGC-1α, PGC-1β and PPARs promote the transcription of β-oxidation- and mitochondrial biogenesis-related genes, enhancing FA oxidation and OXPHOS. |
| ***Cori cycle***  ***-***  ***Lactic acid cycle***  ***-***  ***Lactate-dependent gluconeogenesis*** | The metabolic pathway in which lactate produced by both aerobic and anaerobic glycolysis is transported to the liver and converted to glucose in aerobic conditions, which then returns into the circulatory system. Lactate-dependent gluconeogenesis is also performed in the kidney during adrenergic stimulation. |
| ***Glycolysis*** | The cytoplasmic metabolic pathway that converts glucose into pyruvate as follows:  Glucose + 2 ADP + 2 NAD+ => 2 pyruvates + 2 ATP + 2 NADH + 2 H+  In the presence of O2 along with functional and activated TCA cycle – OXPHOS pathway, aerobic glycolysis is followed by OXPHOS (≈30-33 mol ATP/ mol glucose and high VO2 rates).  In the absence of O2, glycolysis is not followed by OXPHOS (i.e. anaerobic glycolysis ≈2 mol ATP/ mol glucose).  In the presence of O2 along with a high glycolysis / OXPHOS activity ratio, a part of glycolysis is not followed by sufficient OXPHOS (i.e. Warburg-like aerobic glycolysis or aerobic glycolysis ≈2 mol ATP/ mol glucose). |
| ***OXPHOS*** | The metabolic pathway in which cells use nutrient oxidation to release the chemical energy of molecular O_2_ is used to generate ATP from ADP and inorganic phosphate. In all mammals, OXPHOS takes place inside mitochondria.  When the supply of O_2_ is sufficient, this energy comes from feeding pyruvate, one product of glycolysis, into the TCA cycle (i.e. Kreb’s cycle). Pyruvate enters the mitochondria after the conversion to acetyl-CoA. In the TCA cycle, acetyl-CoA is degraded, resulting in NADH,H+ and FADH2 formation, which serve as electron donors for OXPHOS.  Apart from glucose, other sources, such as FA and glutamine, can fuel the TCA cycle. FA are converted into acetyl-CoA via β-oxidation. Glutamine is converted to glutamate via glutaminolysis and enters the TCA cycle after being transformed to into α-ketoglutarate. |
| ***ROS and RNS*** | As a natural by-product of the electron transport within the respiratory chain, ROS are produced alongside OXPHOS, accounting for 1-3 % of total mitochondrial VO_2_ [4,5]. RNS (NO and/or ONOO¯) arise from activation of the inducible NOS isoform (iNOS, NOS 2) to form NO, and the downstream reaction of NO with O_2_^• –^ to form ONOO¯. |
| ***Mitochondria*** | Mitochondria are double-membrane organelles. Cristae formed by the inner membrane contain the four respiratory chain complexes, which oxidize reduced coenzymes (NADH,H+ and FADH2). The resultant electrochemical gradient across the inner membrane triggers ATP synthesis through the FoF1 ATP synthase. However, electron leak occurs throughout complexes I and III, leading to ROS production.  Although mitochondria have their own genetic material (DNA) and membrane structure, they are not independent cellular structures. Their regulation, in terms of amount (biogenesis), shape and network (fusion and fission dynamics), and degradation (mitophagy) is controlled at the scale of an intra-cellular population. Mitochondria constitute a major signaling crossroad that can activate signaling pathways such as apoptosis and inflammation. |
| ***Mitochondrial biogenesis*** | Mitochondrial biogenesis refers to the synthesis of new mitochondrial materials (lipids, proteins, DNA). PGC-1α is the primary transcription factor regulating mitochondrial biogenesis. It promotes expression of other factors such as TFAM, NRFs, ultimately leading to increases in mitochondrial protein synthesis, including respiratory chain subunits, antioxidant enzymes or the DNA polymerase g.  Activations of PGC-1α, PGC-1β and PPARs promote the transcription of β-oxidation- and mitochondrial biogenesis-related genes, enhancing FA oxidation and OXPHOS. |
| ***Mitochondrial DNA*** | The mitochondrial DNA, localized in the matrix, encodes 13 respiratory chain subunits. Although mitochondrial DNA comprises 16,569 base pairs, its genetic background, due to single nucleotide polymorphisms, differs between individuals and constitutes different haplogroups. |
| ***Mitochondrial dynamics*** | Mtochondrial dynamics refer to the mitochondria changes in shapes and networks. Mitochondria are dynamic organelles which can merge (fusion) or divide (fission). Mitofusins and OPA1 control mitochondrial fusion. Mitochondrial fission requires DRP1 and FIS1 [6]. |
| ***Mitochondrial respiration***  ***-***  ***Mitochondrial VO_2_***  ***-***  ***Leak, coupled and uncoupled respiration*** | Mitochondrial respiration refers to mitochondrial VO_2_. In healthy conditions, mitochondrial VO_2_ is predominantly due to the reduction of O_2_ by complex IV of the electron transport chain. More than 95% of electron transport chain VO2 is used to maintain a proton gradient across the inner mitochondrial membrane. The transmembrane electrochemical potential of protons drives to the conversion of ADP and inorganic phosphate into ATP by the F0F1 ATP synthase. Only 1-3 % of total mitochondrial VO2 produces ROS as a natural by-product of the electron transport within the respiratory chain.  Leak respiration refers to the amount of O_2_ consumed by the electron transport chain to compensate for the loss of transmembrane electrochemical potential due to the leak of proton across the inner mitochondrial membrane that is not related to F0F1 ATP synthase activity. Thus, leak respiration is disconnected from ATP synthesis.  Uncoupled mitochondrial respiration refers to mitochondrial respiration associated with high amount of leak respiration (i.e. significantly disconnected from ATP synthesis).  Coupled mitochondrial respiration refers to mitochondrial respiration associated with low amount of leak respiration (i.e. significantly connected to ATP synthesis). |
| ***PPP*** | Metabolic pathway parallel to glycolysis that converts glucose-6 phosphate into five-carbon sugars for nucleotide synthesis, NADPH and FA biosynthesis. Albeit the PPP does involve oxidation of glucose, its primary role is anabolic rather than catabolic. A high flux through aerobic glycolysis subsequently increases the flux through the PPP. |

***ADP***, adenosine diphosphate; ***ATP***, adenosine triphosphate; ***DRP1***, Dynamin related protein 1; ***FA***, fatty acids; ***FADH2***, reduced flavin adenine dinucleotide; ***FIS1***, mitochondrial fission 1 protein; ***H+***, hydrogen ion; ***NADH / NAD+***, nicotinamide adenine dinucleotide in its reduced / oxidized form; ***NADPH***, nicotinamide adenine dinucleotide phosphate; **NRFs**, nuclear respiratory factors; ***OXPHOS***, oxidative phosphorylation; ***NO***, nitric oxide; ***NOS***, NO synthase; ***O_2_***, oxygen; ***O_2_^• –^***, superoxide anion; ***ONOO¯*** - peroxynitrite; ***OPA1***, Optic Atrophy protein 1; ***PGC-1***, PPARγ coactivator-1; ***PPP***, pentose phosphate pathway; ***PPARs***, peroxisome proliferator activated receptors, ***RNS***, reactive nitrogen species; ***ROS***, reactive O_2_ species; ***TCA***, tricarboxylic acid (cycle); ***TFAM***, mitochondrial transcription factor A; ***VO_2_***, O_2_ consumption.

**REFERENCES**

1. Warburg O. On the origin of cancer cells. Science. 24 févr 1956;123(3191):309‑14.

2. Epstein T, Gatenby RA, Brown JS. The Warburg effect as an adaptation of cancer cells to rapid fluctuations in energy demand. PloS One. 2017;12(9):e0185085.

3. Wolfe RR, Herndon DN, Jahoor F, Miyoshi H, Wolfe M. Effect of severe burn injury on substrate cycling by glucose and fatty acids. N Engl J Med. 13 août 1987;317(7):403‑8.

4. Turrens JF. Mitochondrial formation of reactive oxygen species. J Physiol. 15 oct 2003;552(Pt 2):335‑44.

5. Starkov AA. The role of mitochondria in reactive oxygen species metabolism and signaling. Ann N Y Acad Sci. déc 2008;1147:37‑52.

6. Fonseca TB, Sánchez-Guerrero Á, Milosevic I, Raimundo N. Mitochondrial fission requires DRP1 but not dynamins. Nature. 2019;570(7761):E34‑42.
